# High Levels of *β*-Amyloid, Tau, and Phospho-Tau in Red Blood Cells as Biomarkers of Neuropathology in Senescence-Accelerated Mouse

**DOI:** 10.1155/2019/5030475

**Published:** 2019-06-09

**Authors:** Rebecca Piccarducci, Deborah Pietrobono, Carolina Pellegrini, Simona Daniele, Matteo Fornai, Luca Antonioli, Maria Letizia Trincavelli, Corrado Blandizzi, Claudia Martini

**Affiliations:** ^1^Department of Pharmacy, University of Pisa, Pisa, Italy; ^2^Department of Clinical and Experimental Medicine, University of Pisa, Pisa, Italy

## Abstract

Alzheimer's Disease (AD) is the most common Neurodegenerative Disease (ND), primarily characterised by neuroinflammation, neuronal plaques of *β*-amyloid (A*β*), and neurofibrillary tangles of hyperphosphorylated tau. *α*-Synuclein (*α*-syn) and its heteroaggregates with A*β* and tau have been recently included among the neuropathological elements of NDs. These pathological traits are not restricted to the brain, but they reach peripheral fluids as well. In this sense, Red Blood Cells (RBCs) are emerging as a good model to investigate the biochemical alterations of aging and NDs. Herein, the levels of homo- and heteroaggregates of ND-related proteins were analysed at different stages of disease progression. In particular, a validated animal model of AD, the SAMP8 (Senescence-Accelerated Mouse-Prone) and its control strain SAMR1 (Senescence-Accelerated Mouse-Resistant) were used in parallel experiments. The levels of the aforementioned proteins and of the inflammatory marker interleukin-1*β* (IL-1*β*) were examined in both brain and RBCs of SAMP8 and SAMR1 at 6 and 8 months. Brain A*β*, tau, and phospho-tau (p-tau) were higher in SAMP8 mice than in control mice and increased with AD progression. Similar accumulation kinetics were found in RBCs, even if slower. By contrast, *α*-syn and its heterocomplexes (*α*-syn-A*β* and *α*-syn-tau) displayed different accumulation kinetics between brain tissue and RBCs. Both brain and peripheral IL-1*β* levels were higher in SAMP8 mice, but increased sooner in RBCs, suggesting that inflammation might initiate at a peripheral level before affecting the brain. In conclusion, these results confirm RBCs as a valuable model for monitoring neurodegeneration, suggesting peripheral A*β*, tau, and p-tau as potential early biomarkers of AD.

## 1. Introduction

Alzheimer's Disease (AD) is the most common form of Neurodegenerative Disease (ND) and the leading cause of dementia in the elderly population. The molecular hallmarks associated with AD are primarily represented by misfolding and brain deposition of *β*-amyloid protein 1-42 (A*β*), which generates the amyloid plaques, and by neurofibrillary tangles (NTs) of hyperphosphorylated tau protein. According to the “amyloid hypothesis,” A*β* promotes a glycogen synthase kinase-mediated tau phosphorylation, resulting in amyloid plaques and NTs, which damage the blood-brain barrier and produce neuronal apoptosis, inflammation, and oxidative stress [1]. In particular, it has been suggested that neuroinflammation in AD, particularly at its earlier stages, reflects a vicious cycle of microglial activation, release of proinflammatory factors, and neuronal damage [2, 3].

At present, a mixed pattern of protein aggregates in AD and other NDs has been identified [4, 5]. For instance, besides amyloid plaques and NTs, the disease has been associated with intracellular accumulation of *α*-synuclein (*α*-syn) [6], which represents the major component of Lewy bodies and Lewy neuritis, and may play a role in amyloid aggregation in senile plaques [7]. Interestingly, *α*-syn has been shown to physically interact with tau [8, 9] or A*β* [8] giving rise to the formation of hybrid proteins (“heteroaggregates”) in brains of patients affected by NDs [4, 5, 10].

Brain amyloid accumulation and aggregation have been shown to kick off even decades before the onset of clinical disease symptoms, and to reach the biological compartments. In this context, cerebrospinal fluid (CSF) has proven the most explored medium, as it is deemed to reproduce brain pathological processes [5, 11]. Consistently, AD diagnosis is nowadays based on the identification of misfolded-aggregated proteins in the brain (where amyloid plaques are visualised through imaging techniques) and CSF [12, 13]. However, considering the disadvantages that limit the clinical use of CSF, several efforts have been devoted to explore fluids other than CSF as a source of neurodegeneration biomarkers. In this respect, it has been suggested that pathological alterations of blood proteins reflect the changes in CSF due to the simple diffusion or barrier impairment that characterises neurodegeneration [11, 14].

Red Blood Cells (RBCs) are emerging as a good model to investigate the biochemical alterations related to aging and neurodegeneration, including oxidative stress and inflammation [15, 16]. Indeed, RBCs are highly susceptible to oxidative damage, due to the high concentration of oxygen and haemoglobin [17, 18]. Moreover, recent findings have shown an accumulation of ND-related proteins in these cells and its relationship with NDs [11, 19–21]. In particular, the presence of misfolded proteins (*α*-syn, A*β*, and tau) and their aggregates (*α*-syn-A*β* and *α*-syn-tau) has been recently proved in RBCs from healthy subjects and patients with NDs [5, 10, 11, 22]. At present, putative correlation between the accumulation of the aforementioned proteins in the brain and RBCs and inflammation has never been investigated.

To this end, we used here an animal model of AD, SAMP8 (Senescence-Accelerated Mouse-Prone), which is characterised by an early beginning of irreversible and severe learning and memory deficits. At a molecular level, this animal model displays an increase in A*β* proteins in hippocampal granules, hyperphosphorylation of tau protein, increase in *α*-synuclein, and increase in oxidative damage [23]. SAMR1 (Senescence-Accelerated Mouse-Resistant), which does not develop the disease [23], was used as control.

Herein, misfolded proteins were quantified in both the brain and RBCs of SAMP8 and SAMR1 mice, in order to establish a correlation between brain and peripheral fluids, as well as to ascertain the putative use of misfolded proteins in RBCs as AD biomarkers. Finally, to establish the link between brain/peripheral inflammation and accumulation of ND-related proteins, interleukin-1*β* (IL-1*β*) was quantified as one representative marker [14]. Indeed, neuroinflammatory cytokines, including IL-1*β*, have been involved in the formation of AD neuritic plaques [24–26] and were found in higher quantities in AD with respect to controls in both humans and animals [27–29].

## 2. Materials and Methods

### 2.1. Animals

SAMP8 mice (2 months old, 20-25 g body weight), employed as a spontaneous genetic model of AD [30], and their control strain SAMR1 (2 months old, 20-25 g body weight) were purchased from Envigo SRL (San Pietro al Natisone UD, Italy).

Animal care and handling were in accordance with the provisions of the European Community Council Directive 210-63-EU, recognised and adopted by the Italian Government. The experiments were approved by the Ethical Committee for Animal Experimentation of the University of Pisa and by the Italian Ministry of Health (authorization no. 198-2016-PR). One day after the end of cognitive tests (see the following discussion), the animals were euthanized by cervical dislocation and the cerebral cortex was carefully isolated by microscopic forceps. Blood samples were collected by cardiac puncture.

In the acquisition training, animals are subjected to sessions of four trials every day for 2 days. In the hidden-platform training, performed by submerging the platform 1.5 cm below the surface of the water, animals are subjected to sessions of four trials every day for 5 days. Finally, in the probe trial on the eighth day, the platform is removed and the number of target crossings, number of entries into the target quadrant, and the time spent in the target quadrant are assessed as measures for 60 s.

### 2.2. Evaluation of Cognitive Functions: Morris Water Maze Test and Behavioural Test

**Figure 1:**
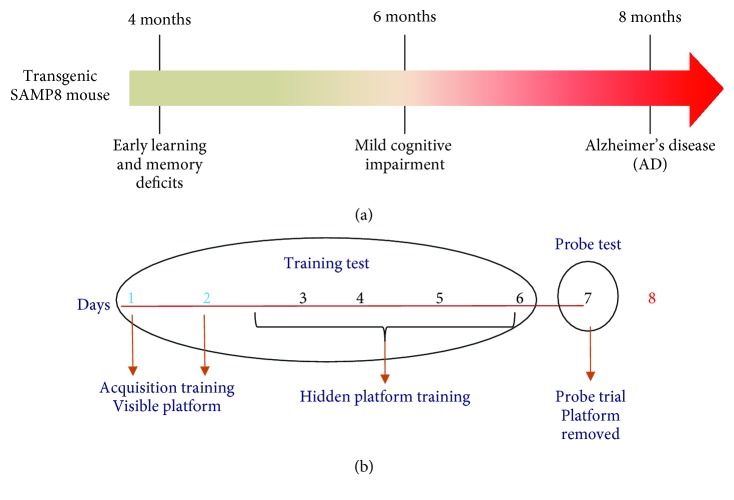
(a) Diagram showing the timing of cognitive impairment and AD progression in SAMP8 mice. (b) Diagram showing the different phases of the behavioural test.

The behavioural test consists of visible-platform acquisition training, hidden-platform training, and probe trial (Figure 1). The platform was in the same location for both visible-platform training and hidden-platform training. In the acquisition training, the escape latency was assessed for each animal (time required to reach the platform). Mice were placed on the platform for 10 s before being released into the water. Mice were allowed to swim and find the visible platform within 60 s. Each animal was subjected to sessions of four trials every day for 2 days. After the daily trial, mice were returned to their home cages for resting. In the hidden-platform training, performed by submerging the platform 1.5 cm below the surface of the water, escape latency was evaluated over the next 5 days (Figure 1). Each animal was subjected to sessions of four trials every day. Finally, on the eighth day, the platform was removed from the tank for the probe trial (Figure 1). The number of target crossings, number of entries into the target quadrant, the time spent in the target quadrant where the platform was placed, the swimming speed, swim distance, and swim distance in the target quadrant were assessed as measures for 60 s. Data are expressed as raw values, while the data regarding the time spent in the target quadrant are expressed as the percent time spent in the quadrant with the platform in comparison to each of the other quadrants.

### 2.3. Collection of Brain Tissues and RBCs

Brains were dissected from mice. The samples were suspended in phosphate-buffered saline (PBS) and then sonicated. The blood was sampled from mice, and it was preserved into an EDTA tube. A centrifugation at 200 × g at 4°C for 10 min was required to isolate RBCs from plasma. The RBC pellet was suspended in 3 ml of PBS and subjected to centrifugation at 1000 × g for 10 min and washed thrice with PBS. Finally, RBCs were centrifuged at 1500 × g for 10 min and frozen at -20°C until use.

### 2.4. Coimmunoprecipitation-Western Blotting

RBC (1 mg) and brain (30 *μ*g) samples were lysed in RIPA buffer and later resolved by SDS-PAGE (8.5%) to assess the expression of A*β*, *α*-syn, and tau. Samples were transferred to PVDF membranes and probed overnight at 4°C with primary antibodies to A*β* (*β*-amyloid H-43, sc-9129, Santa Cruz Biotechnology Inc.), *α*-syn (*α*-*β* synuclein N-19, sc-7012, Santa Cruz Biotechnology Inc.), or tau (H-150, sc-5587, Santa Cruz Biotechnology Inc.) [11, 22]. Then, the primary antibodies were revealed through peroxidase-conjugated secondary antibodies and a chemiluminescent substrate (ECL, PerkinElmer).

A coimmunoprecipitation assay was used to prove the presence of *α*-syn heterocomplexes with A*β* or tau [11, 22]. Briefly, lysates (1 mg) recovered from RBCs or the brain were suspended in RIPA buffer; they were probed with an anti-*α*-syn antibody (5 *μ*g sample), maintained under constant rotation overnight, and successively immunoprecipitated with protein A-Sepharose. The immunocomplexes were suspended in Laemmli solution following an extensive washing; thereafter, the immunocomplexes were determined by SDS-PAGE and probed overnight with primary antibodies to *α*-syn (input), A*β*, or tau as described previously [11, 22].

### 2.5. Preparation of Aged Solutions of *α*-Syn and of the *α*-Syn-Biotinylated Antibody

The incubation of recombinant *α*-syn took place in parafilm-sealed tubes at 37°C for 4 days in an Eppendorf ThermoMixer under continuous mixing (1000 rpm) [31]. The *α*-syn-biotinylated antibody was prepared through a reaction among Sulfo-NHS-LC-Biotin (Pierce, Rockford, IL, USA) (200 mg) and the 211 mouse monoclonal antibody (mAb) (Santa Cruz Biotechnology Inc., Santa Cruz, CA, USA) [32]. To remove excess uncoupled biotin, the mixture was desalted on Bio-Spin-6 Columns (Bio-Rad, UK) [11, 22].

### 2.6. Immunoassay Methods

The presence and quantification of total A*β*, tau, and *α*-syn and its heterocomplexes (*α*-syn-A*β* and *α*-syn-tau) in RBCs and in brain tissues were assessed by a “homemade” sandwich enzyme-linked immunosorbent assay (ELISA) system [11, 33, 34]. The plate was precoated overnight with 60 *μ*l-well of a specific antibody directed to the protein in analysis. Following an extensive washing with PBS-T (PBS, containing 0.01% Tween 20), BSA 1% (200 *μ*l-well) was added to block nonspecific sites and the plate was incubated. After additional washes with PBS-T, RBCs and brain tissues were added to each well (100 *μ*l-well) and incubated. Following an extensive washing, an antibody (75 *μ*l-well) directed to a specific amino-acidic sequence of the protein was employed for capturing, followed by an incubation. For antigen detection, an HRP antibody (100 *μ*l-well) was used and it was incubated [11]. The wells were then washed with PBS-T before the addition of 100 *μ*l-well of 3,3′,5,5′-tetramethylbenzidine (TMB) (Thermo Fisher Scientific). The absorbance was evaluated at 450 nm after the addition of the Stop Solution (0.4 N HCl, 100 *μ*l-well). All measurements were performed in duplicate to reduce interassay variability. The standard curve for the ELISA assay was constructed using a recombinant human protein solution at different concentrations diluted in PBS [11, 22, 34]. The concentration of ND-related proteins in the brain and RBCs was expressed as the nanogram or picogram of protein and normalised to the quantity of total proteins present in the analysed samples.

#### 2.6.1. Detection of Total *α*-Synuclein

The plate was coated with full-length polyclonal antibody to *α*-syn (sc-10717, Santa Cruz Biotechnology Inc.) overnight at 4°C. After the incubation with BSA 1% and three washes with PBS-T, RBCs (0.1 mg-100 *μ*l) and brain tissues (10 *μ*g-100 *μ*l) were added to each well and incubated at 25°C for 2 h. Then, mouse monoclonal antibody to *α*-syn (sc-12767, Santa Cruz Biotechnology Inc.) was employed and incubated at 37°C for 2 h. An anti-mouse-HRP antibody was used, and it was incubated at 37°C for 1.5 h [11, 21, 22, 35].

#### 2.6.2. Detection of Total A*β*

A precoating with a specific antibody to A*β* (sc-9129, Santa Cruz Biotechnology Inc.) was prepared (60 *μ*l-well) and maintained overnight at 4°C. BSA 1% was added and the plate was incubated for 2 h at 37°C. RBCs (0.05 mg-100 *μ*l) and brain tissues (0.25 *μ*g-100 *μ*l) were added to each well and incubated at 25°C for 1 h. Samples were probed with polyclonal antibody to A*β* (sc-5399, Santa Cruz Biotechnology Inc.) (75 *μ*l-well) for 1.5 h at 25°C. For antigen detection, a donkey anti-goat-HRP antibody was incubated at 37°C for 1 h [11, 32, 36].

#### 2.6.3. Detection of Total Tau

The plate was precoated with a specific antibody to tau (sc-32274, Santa Cruz Biotechnology Inc.) and left overnight at 4°C. BSA 1% was added and the plate was incubated for 1 h at 37°C. RBCs (0.4 mg-100 *μ*l) and brain tissues (2 *μ*g-100 *μ*l) in each well were incubated at 25°C for 2 h. Samples were probed with polyclonal antibody to tau (sc-5587, Santa Cruz Biotechnology Inc.) and incubated at 37°C for 2 h. A goat anti-rabbit-HRP antibody (Invitrogen) was incubated for 1.5 h [11, 32, 36].

#### 2.6.4. Detection of Phospho-Tau

The plate was precoated with a specific antibody to tau (sc-32274, Santa Cruz Biotechnology Inc.) and left overnight at 4°C. BSA 1% (200 *μ*l-well) was added, and the plate was incubated for 2 h at 37°C. RBCs (0.2 mg-100 *μ*l) and brain tissues (1 *μ*g-100 *μ*l) in each well were incubated at 25°C for 2 h. Samples were probed with polyclonal antibody to tau (70R-32555, Fitzgerald Industries International) and incubated at 37°C for 1.5 h. For antigen detection, goat anti-rabbit-HRP antibody (Invitrogen) was incubated for 1.5 h [11, 32].

#### 2.6.5. Immunoassay Detection of *α*-Syn-Tau Heterocomplexes

The ELISA plate was coated with *α*-*β*-syn N-19 antibody (sc-7012, Santa Cruz Biotechnology Inc.) overnight at room temperature. RBCs (400 *μ*g-100 *μ*l) and brain tissues (1 *μ*g-100 *μ*l) were added to each well and incubated at 25°C for 2 h. BSA 1% was added to each well for 20 min at 37°C. Rabbit polyclonal anti-tau H-150 antibody (sc-5587, Santa Cruz Biotechnology Inc., 37°C for 2 h) was employed for capturing. A goat anti-rabbit-HRP antibody was used at 37°C for 1.5 h [11]. The concentration of *α*-syn-tau interaction in the samples was quantified according to a standard curve [11], which was constituted using a solution of recombinant human *α*-syn and recombinant human tau at different concentrations in SDS 2 mM. The solution was prepared by incubating 1 mg of each protein, diluted in 2 mM SDS, in parafilm-sealed tubes at 37°C for 1 h in an Eppendorf ThermoMixer with continuous mixing (500 rpm) [11, 22].

#### 2.6.6. Immunoassay Detection of *α*-Syn-A*β* Heterocomplexes

The ELISA plate was coated with *β*-amyloid H-43 antibody (sc-9129, Santa Cruz Biotechnology Inc.) overnight at room temperature. RBCs (0.2 mg-100 *μ*l) and brain tissues (1 *μ*g-100 *μ*l) were added to each well and incubated at 25°C for 2 h. BSA 1% was incubated for 20 minutes at 37°C. A mouse monoclonal anti-*α*-synuclein 211 antibody (sc-12767, Santa Cruz Biotechnology Inc.) was employed for capturing and was incubated at 37°C for 2 h. Goat anti-mouse-HRP antibody was incubated at 37°C for 1.5 h [11]. The concentration of *α*-syn-A*β* interaction in the samples was quantified according to a standard curve [11], which was constituted using a solution of recombinant human *α*-syn and recombinant human A*β* at different concentrations in SDS 2 mM. The solution was prepared by incubating 1 mg of each protein, diluted in 2 mM SDS, in parafilm-sealed tubes at 37°C for 16 h in an Eppendorf ThermoMixer with continuous mixing (500 rpm) [11, 37].

### 2.7. Evaluation of IL-1*β* Levels in Brain Tissues and in RBCs

IL-1*β* levels in the cerebral cortex and RBCs were measured by a commercial enzyme-linked immunosorbent assay kit (Abcam, Cambridge, UK), as previously described. Briefly, 20 mg of cerebral cortex, stored previously at -80°C, were homogenised in 400 *μ*l of PBS, pH 7.2, at 4°C and centrifuged at 10000 × g for 5 min. Aliquots (50 *μ*l) of the supernatants were then used for the assay. In parallel, 0.2 mg-50 *μ*l of RBCs, homogenised in PBS, were used for the assay. IL-1*β* levels were expressed as picogram per milligram of total proteins present in the analysed samples.

### 2.8. Statistical Analysis

The results are presented as the mean ± S.E.M. unless otherwise stated. The significance of differences was evaluated by a two-way analysis of variance followed by post hoc analysis with the Fisher LSD test or a one-way analysis of variance followed by post hoc analysis with the Student-Newman-Keuls test where appropriate. *P* values <0.05 were deemed significantly different. All statistical procedures were performed by commercial software (GraphPad Prism, version 7.0; GraphPad Software Inc., San Diego, CA).

## 3. Results

### 3.1. Evaluation of Cognitive Functions (Morris Water Maze Test)

**Figure 2:**
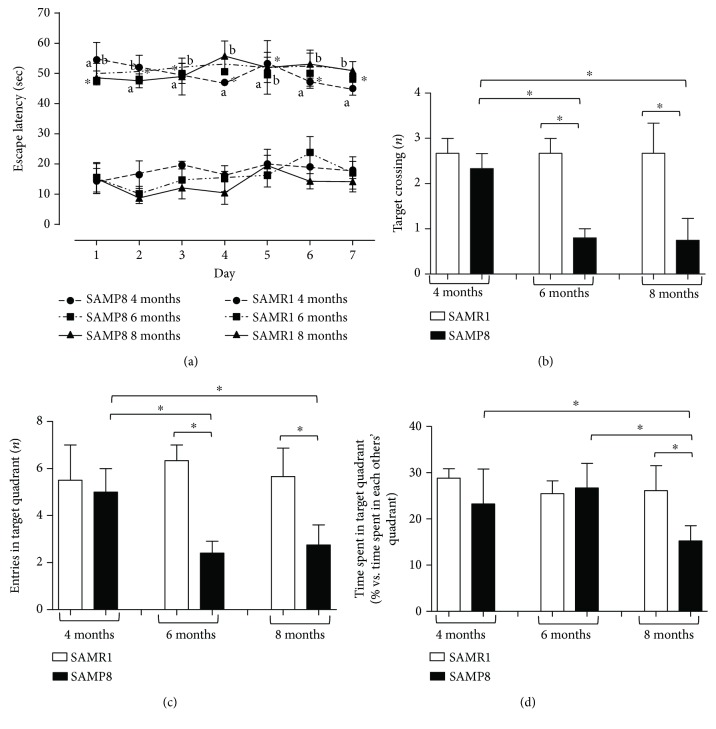
Cognitive performance of SAM. (a) Escape latency in SAMR1 and SAMP8 mice at 4, 6, and 8 months of age, during seven consecutive days of Morris water maze test training. Cognitive performance of SAMR1 and SAMP8 mice at 4, 6, and 8 months of age, during the probe trial session of the Morris water maze test; (b) number of target crossings; (c) entries into the target quadrant; (d) time spent within the target quadrant. Data are expressed as mean ± S.E.M. obtained from 8 animals. Differences among groups were evaluated by two-way analysis of variance followed by post hoc analysis with the Fisher LSD test or a one-way analysis of variance followed by post hoc analysis with the Student-Newman-Keuls test where appropriate. ^∗^*P* < 0.05 between the indicated subgroups.

As a first step, mice cognitive functions, i.e., spatial learning and memory ability, were evaluated by the Morris water maze test. SAMR1 mice rapidly learned the location of the platform (Figure 2(a)). SAMP8 mice at 4, 6, and 8 months displayed a significant increase in escape latency time at every test day compared with SAMR1 mice (Figure 2(a)).

These findings are in line with other studies showing that SAMP8 mice displayed a significant increase in escape latency time since the first day of training as compared with SAMR1 mice, while no significant difference was observed among training days [38, 39].

**Figure 3:**
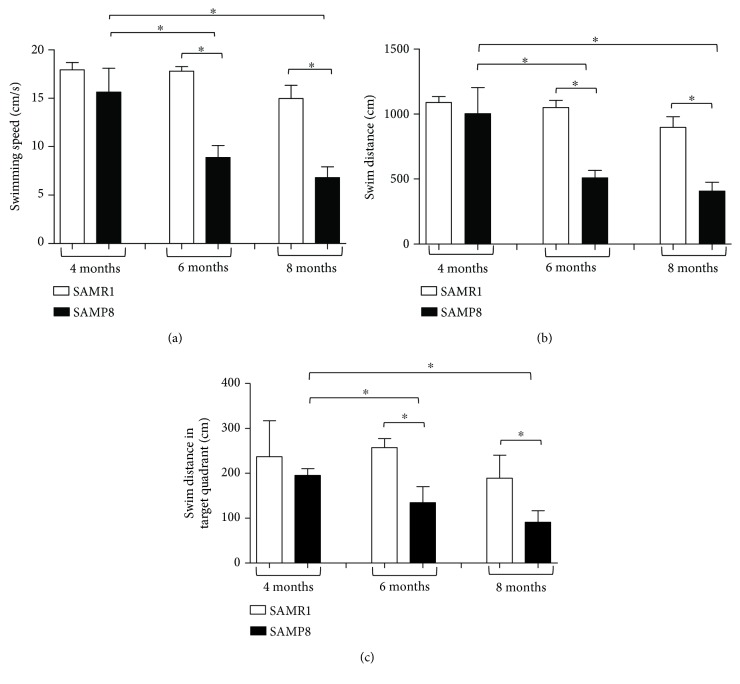
Cognitive performance of SAM. Cognitive performance of SAMR1 and SAMP8 mice at 4, 6, and 8 months of age, during the probe trial session of the Morris water maze test: (a) swimming speed; (b) swim distance; (c) swim distance in the target quadrant. Data are expressed as mean ± S.E.M. obtained from 8 animals. Differences among groups were evaluated by two-way analysis of variance followed by post hoc analysis with the Fisher LSD test or a one-way analysis of variance followed by post hoc analysis with the Student-Newman-Keuls test where appropriate. ^∗^*P* < 0.05 between the indicated subgroups.

During the probe trial, the number of target crossings as well as the entries into the target quadrant were significantly decreased in SAMP8 mice at 6 and 8 months, compared with respective controls (SAMR1) and SAMP8 animals at 4 months (Figures 2(b) and 2(c)). The time spent within the target quadrant was significantly decreased in SAMP8 mice at 8 months, compared with respective SAMR1 animals and SAMP8 mice at 4 and 6 months (Figure 2(d)). Considering the swimming speed, the swim distance, and the swim distance in the target quadrant, our results show that SAMP8 animals displayed a decrease in swimming speeds and distance traveled, as compared with age-matched SAMR1 (Figures 3(a)–3(c)). Of note, our results point out that SAMP8 animals did not float in the quadrant where the platform was located, but they reached it slowly, thus excluding despair or depression signs. In addition, the number of target crossings and the entries into the target quadrant were significantly decreased in SAMP8 mice starting from 6 months of age as compared with age-matched SAMR1, while the time spent in the target quadrant decreased significantly in SAMP8 animals at 8 months of age. This discrepancy could be ascribed to a learning and memory deficit in looking for the platform and to a decrease in swimming speed, as well as to a reduced motivation to escape from the water of SAMP8 mice not being in an aversive situation [40].

Altogether, these data confirm that SAMP8 mice develop a deficit in spatial learning and memory performance compared with control SAMR1 mice. Moreover, such deficits become clearer as AD develops in SAMP8 mice.

### 3.2. Expression of *α*-Syn, Tau, and A*β* in Brain Tissues and RBCs: Immunoblotting Analysis

The presence of AD-related misfolded proteins (i.e., *α*-syn, tau, and A*β*) was assessed by western blotting analysis in brain and RBC samples obtained from SAM mice. The quantitative comparison between pathological and control animals was subsequently performed by immunoenzymatic assays. Considering the timing of ageing, the demonstrated cognitive impairment, and the onset of the pathological processes, samples obtained from 4-month animals were excluded from further investigations, focusing on the brain tissues and RBCs obtained from animals at 6 and 8 months.

**Figure 4:**
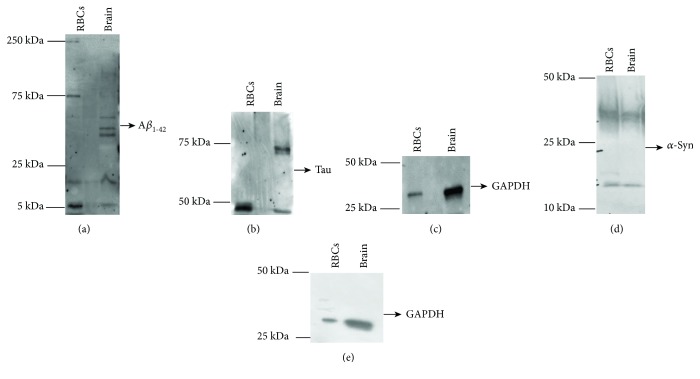
Detection of misfolded proteins in cell lysates of RBCs and brain tissues by western blot analysis. Cell lysates of RBCs and brain tissues from SAMs were immunoblotted with antibodies to A*β* (a), tau (b), and *α*-syn (d). (c, e) GAPDH was used as a loading control for A*β* and tau (c) or for *α*-syn (e) normalisation. A representative image for each protein is shown (*n* = 3).

The anti-A*β* antibody recognised 5 and 15 kDa proteins (Figure 4(a)) corresponding to A*β* monomeric and oligomer forms, respectively [41], in both brain tissues and RBCs. Bands with a molecular weight higher than 25 kDa (Figure 4(a)) were especially revealed in brain tissues, presumably indicating elevated oligomeric forms of the protein [42].

In brain tissues, the anti-tau antibody revealed the characteristic bands (Figure 4(b)) ranging from 55 to 74 kDa [11, 43, 44]. Worth noting, a band lower than 50 kDa, which was particularly evident in RBCs (Figure 4(b)), has been related to truncated or cleaved forms of tau containing the C-terminal region [11, 43, 44]. These data demonstrate that the antibody is able to recognise both truncated and uncleaved forms of the protein and that the triplet bands do not refer to oligomeric tau.

Finally, the anti-*α*-syn antibody-labelled proteins of 15 kDa and 30 kDa proteins correspond to *α*-syn [43] in both brain tissues and RBCs (Figure 4(d)).

### 3.3. Expression of *α*-Syn Heterocomplexes with Tau or A*β* in Brain Tissues and RBCs of SAM Mice

**Figure 5:**
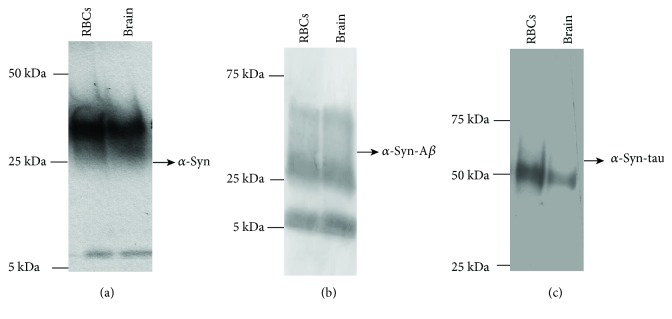
Detection of *α*-syn heterocomplexes with tau and A*β* in RBCs and brain samples. From SAMs, cell lysates of RBCs and brain tissues were immunoprecipitated with an anti-*α*-syn antibody and then immunoblotted with antibodies to *α*-syn ((a), i.e., input), A*β* (b), or tau (c). A representative image for each protein is shown.

A coimmunoprecipitation-western blotting assay was employed to explore the presence of *α*-syn heterocomplexes in brain and RBC samples of SAM mice (Figure 5). To this purpose, lysates obtained from brain tissues or RBCs were immunoprecipitated using an anti-*α*-syn antibody and then immunoblotted with a specific antibody for *α*-syn (i.e., input), tau, or anti-A*β* [11]. In *α*-syn immunoprecipitates (Figure 5(a)), two major bands of 15 kDa and 30 kDa, corresponding to *α*-syn protein [11, 45], were noticed in both brain tissues and RBCs.

The A*β* immunoblotting on *α*-syn immunoprecipitates obtained from brain tissues and RBCs produced two main immunoreactive bands of 5 and 30 kDa (Figure 5(b)), mainly associated to monomeric and oligomeric A*β* forms [11]. Moreover, probing *α*-syn immunoprecipitates with an anti-tau antibody showed an immunoreactive band of 55 kDa, ascribable to the tau protein (Figure 5(c)). Altogether, the results demonstrated that *α*-syn forms heterocomplexes with A*β* and tau in brain tissues and in RBCs of SAM mice, similarly to previous data reported in human samples [5, 11, 22].

### 3.4. Concentrations of *α*-Syn, Tau, and A*β* in Brain Tissues and RBCs of SAM Mice

Specific and quantitative immunoenzymatic assays were employed to measure *α*-syn, tau, and A*β* levels in brain tissues and RBCs of SAMP8 mice at different stages of AD progression and their age-matched SAMR1 controls. The values are set out in Table 1.

#### 3.4.1. A*β*

**Figure 6:**
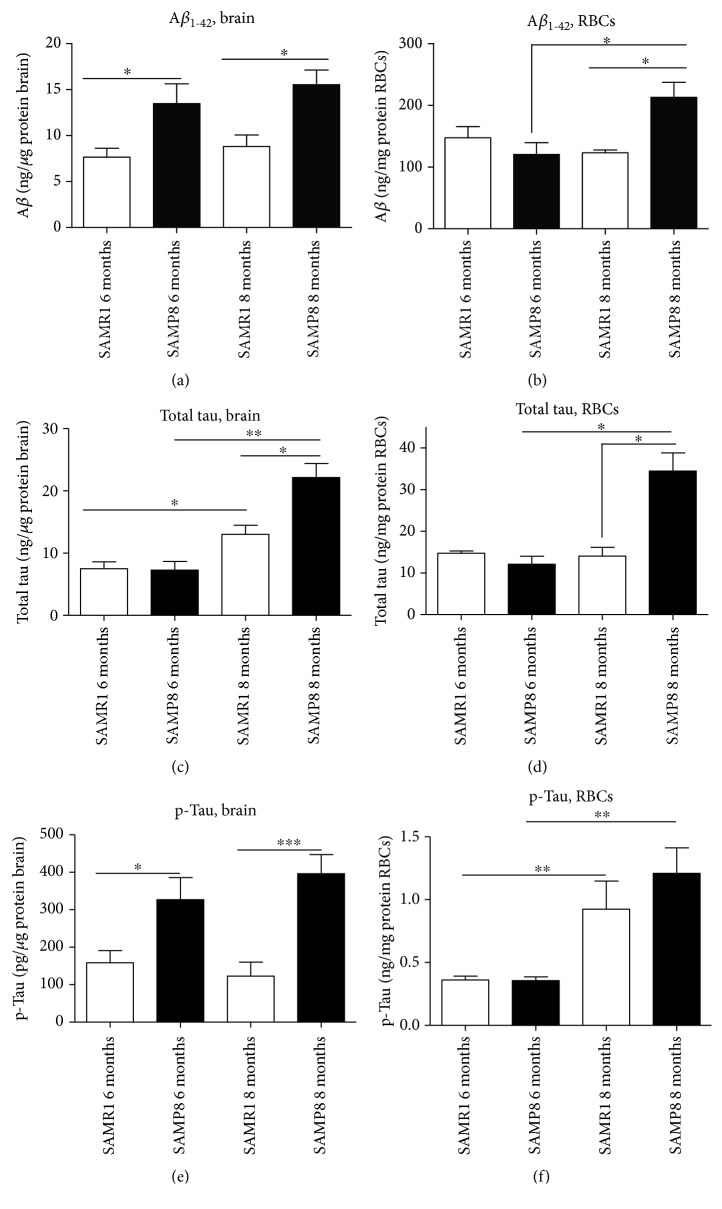
Quantitative assay of misfolded protein A*β*, tau, and p-tau in RBCs and brain tissues. The levels of A*β*, tau, and p-tau in brain tissues (a, c, and e) and RBCs (b, d, and f) from SAMP8 and SAMR1 mice at 6 and 8 months were assessed by specific immunoenzymatic assays. Differences among groups were evaluated by one-way ANOVA. *P* values were adjusted with Sidak's multiple comparison test: ^∗^*P* < 0.05, ^∗∗^*P* < 0.01, and ^∗∗∗^*P* < 0.001 between the indicated subgroups.

The amount of A*β* in the brain did not change with age either in SAMR1 or in SAMP8 (Figure 6(a)), although an incremental trend was noticed. By contrast, SAMP8 animals showed significantly higher A*β* concentrations than age-matched SAMR1 mice (6 months: *P* = 0.0193; 8 months: *P* = 0.0104), consistent with previous data. These results confirm that A*β* accumulated early in the brain of AD animals.

As observed in the brain, the levels of A*β* in RBCs of SAMR1 did not change with age (Figure 6(b)). By contrast, A*β* significantly accumulated with age in RBCs from SAMP8 (*P* = 0.0270, Figure 6(b)). Moreover, SAMP8 animals at 8 months displayed significantly higher A*β* RBC levels than the age-matched SAMR1 (*P* = 0.0279), consistent with the data obtained in the brain. By contrast, comparable levels were noticed in SAMP8 and SAMR1 at 6 months (*P* = 0.3029, Figure 6(b)). These data suggest that A*β* accumulated in RBCs with AD progression, albeit with a slower accumulation kinetics than that observed in the brain.

#### 3.4.2. Tau

Brain levels of total tau (Figure 6(c)) were proven to increase significantly with age either in SAMR1 (*P* = 0.0293) or in SAMP8 mice (*P* = 0.0095). Moreover, SAMP8 animals at 8 months displayed significantly higher brain concentrations of total tau compared to their age-matched controls (*P* = 0.0350). These data confirm that tau accumulated in the brain along with AD progression in the SAM experimental model.

Tau concentrations in RBCs increased with age in SAMP8 only (*P* = 0.0019; SAMR1: *P* = 0.7430, Figure 6(d)). Consistent with the data obtained in the brain, total tau significantly accumulated in RBCs of SAMP8 mice at 8 months compared to control mice (*P* = 0.00019, Figure 6(d)). These results suggest that tau accumulation in RBCs in pathological mice may reflect the increase of this protein in the brain.

#### 3.4.3. Phospho-Tau

The levels of p-tau in the brain (Figure 6(e)) did not differ with mice ages, either in SAMP8 or in SAMR1 (*P* = 0.4140 and *P* = 0.5737, respectively, Figure 6(e)). By contrast, pathological mice displayed significantly higher levels of brain p-tau compared to the age-matched controls, either at 6 months or at 8 months (*P* = 0.0485 and *P* = 0.0010, respectively, Figure 6(e)). These data confirm that tau is hyperphosphorylated in the brain of the AD animal model.

Interestingly, an age-dependent p-tau accumulation was observed in RBCs, both in control (*P* = 0.0011, Figure 6(f)) and SAMP8 mice (*P* = 0.0015, Figure 6(f)). Nevertheless, no significant changes in p-tau levels in RBCs were observed comparing SAMP8 and SAMR1 (6 months: *P* = 0.6965; 8 months: *P* = 0.09401, Figure 6(f)). These results suggest that phosphorylated tau accumulated in RBCs both under physiological ageing and AD progression.

#### 3.4.4. *α*-Syn

**Figure 7:**
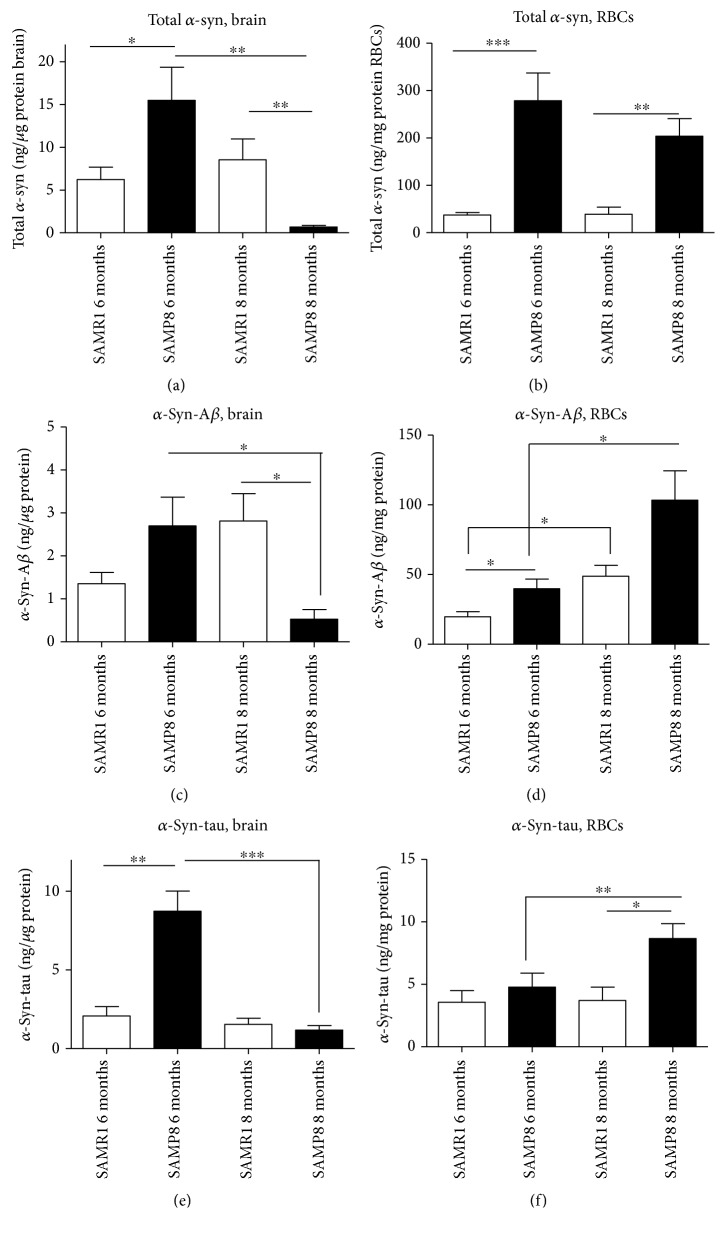
Quantitative assay of *α*-syn and misfolded protein's heterocomplex in both RBCs and in brain tissues. Levels of *α*-syn, *α*-syn-A*β*, and *α*-syn-tau in brain tissues (a, c, and e) and in RBCs (b, d, and f) from SAMP8 and SAMR1 mice at 6 and 8 months were assessed by specific immunoenzymatic assays. Difference among groups were evaluated by one-way ANOVA. *P* values were adjusted with Sidak's multiple comparison test: ^∗^*P* < 0.05, ^∗∗^*P* < 0.01, and ^∗∗∗^*P* < 0.001 between the indicated subgroups.

The brain concentrations of total *α*-syn significantly decreased with animals' age in SAMP8 (*P* = 0.0095, Figure 7(a)), but not in control animals (*P* = 0.5622, Figure 7(a)), suggesting that total *α*-syn decreases as the disease progresses. Consistent with these data, SAMP8 mice at 8 months presented significantly lower brain concentrations of total *α*-syn compared to age-matched controls (*P* = 0.0022, Figure 7(a)). Surprisingly, the opposite condition was found when comparing animals at 6 months (*P* = 0.0240, Figure 7(a)).

Total *α*-syn in RBCs did not show significant variations with age (SAMR1: *P* = 0.2643, Figure 7(b)) or AD progression (SAMP8: *P* = 0.8968, Figure 7(b)). Differently from the brain, RBCs from SAMP8 animals showed significantly higher total *α*-syn concentrations compared to the age-matched controls (6 months: *P* < 0.0001; 8 months: *P* = 0.0097, Figure 7(b)). Globally, these data suggest a different kinetics of *α*-syn in peripheral fluids compared to the brain.

### 3.5. Concentrations of *α*-Syn Heterocomplexes in Brain Tissues and RBCs

*α*-Syn heterocomplexes with A*β* or tau were measured both in brain tissues and RBCs through a “homemade” immunoenzymatic assay [11, 22].

#### 3.5.1. *α*-Syn-A*β*

The levels of *α*-syn-A*β* in SAMP brains (Figure 7(c)) showed an interesting trend depending on ageing and progression of the disease. Indeed, the brain concentrations decreased along with age-AD progression in SAMP8 mice (*P* = 0.0303, Figure 7(c)). Moreover, *α*-syn-A*β* levels in the brain decreased in SAMP8 at 8 months compared to age-matched controls (*P* = 0.0101, Figure 7(c)). These data suggest that brain *α*-syn-A*β* levels may follow the accumulation kinetics of total *α*-syn.

Surprisingly, *α*-syn-A*β* levels in RBCs increased with age-AD progression in SAMR1 (*P* = 0.0017, Figure 7(d)) and SAMP8 mice (*P* = 0.0308). Moreover, such levels in RBCs were higher in SAMP8 compared to age-matched controls, even if a statistical significance was reached at 6 months only (6 months: *P* = 0.0168; 8 months. *P* = 0.1797, Figure 7(d)). These data suggest that the *α*-syn-A*β* level in RBCs has an opposite trend compared to that in the brain.

#### 3.5.2. *α*-Syn-Tau

The levels of *α*-syn-tau heterocomplexes in the brain decreased with the pathological progression in SAMP8 mice (*P* = 0.0002, Figure 7(e)). Nevertheless, such concentrations were significantly elevated in SAMP8 compared to SAMR1 at 6 months (*P* = 0.0012, Figure 7(e)). Taken together, these data suggest that the *α*-syn-tau trend in the brain reflects what is elicited by *α*-syn.

The levels of *α*-syn-tau heterocomplexes in RBCs did not significantly differ in SAMR1 at different ages (*P* = 1.000, Figure 7(f)) or between SAMR1 and SAMP8 at 6 months (*P* = 0.8907, Figure 7(f)). By contrast, *α*-syn-tau concentrations increased in SAMP8 mice at 8 months compared to the age-matched controls (*P* = 0.0155, Figure 7(f)) or to SAMP8 at 6 months (*P* = 0.0025, Figure 7(f)). These data suggest that the *α*-syn-tau level in RBCs has an opposite trend compared to that in the brain.

### 3.6. Assessment of IL-1*β* Levels in Brain Tissues and RBCs

In order to monitor the progress of inflammation in the animal model of AD, IL-1*β* was chosen as a representative inflammatory factor [14] and measured in brain tissues of SAMP8 mice and in their age-matched SAMR1 controls.

**Figure 8:**
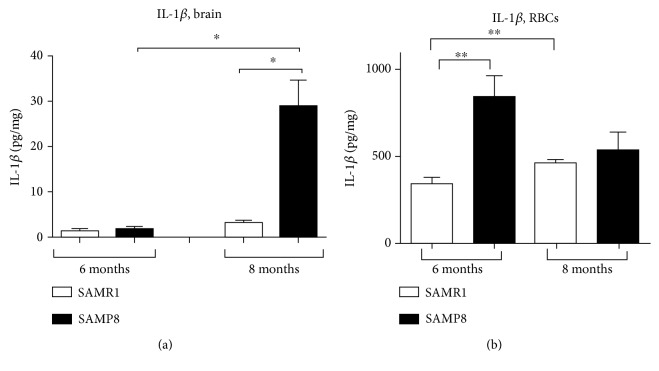
Quantitative detection of IL-1*β*. IL-1*β* levels were assessed in brain (a) and RBC (b) tissues from control animals (SAMR1) and SAMP8 mice at 6 and 8 months of age. Each column represents the mean ± S.E.M. value (*n* = 8). Differences among groups were evaluated by one-way ANOVA. *P* values were adjusted with Sidak's multiple comparison test: ^∗^*P* < 0.05 and ^∗∗^*P* < 0.01 between the indicated subgroups.

In the cerebral cortex from SAMR1 mice at 6 and 8 months, IL-1*β* levels accounted for 1.3 ± 0.5 and 3.3 ± 0.4 pg − mg, respectively (Figure 8(a)), suggesting an increase in interleukin levels with physiological ageing. At 6 months, SAMP8 mice showed IL-1*β* levels of 1.8 ± 0.7 pg − mg in the cerebral cortex (Figure 8(a)), which were comparable to those detected in the respective age-matched control (SAMR1) mice. By contrast, IL-1*β* levels in the cerebral cortex from SAMP8 mice at 8 months were significantly increased (29.6 ± 5.4 pg − mg) compared to the age-matched controls (SAMR1) and to SAMP8 mice at 6 months (Figure 8(a)). These data suggest that brain IL-1*β* concentrations increased in the AD animal model and that such an increase became more prominent with age (i.e., with AD progression) in SAMP8 mice compared with aged SAMR1 mice.

In order to check whether brain inflammation was associated with an enhancement in peripheral inflammatory factors, IL-1*β* concentration was monitored in RBCs, which are able to produce cytokines, too [46]. The interleukin significantly accumulated with ageing (6 months vs. 8 months) in RBCs of SAMR1 mice (Figure 8(b)). Surprisingly, an opposite trend was observed in SAMP8 mice (Figure 8(b)), which displayed the highest IL-1*β* concentrations at 6 months (839.7 ± 398.1 pg − mg, Figure 8(b)). The IL-1*β* concentration in RBCs was significantly higher in SAMP8 mice at 6 months compared to the age-matched control (i.e., SAMR1, 6 months, 384.4 ± 72.98 pg − mg); by contrast, comparable values were obtained in SAMP8 and SAMR1 at 8 months (538.8 ± 421.2 and 460.9 ± 42.12 pg − mg, Figure 8(b)). Overall, these data show that the inflammatory cytokine is released maximally in RBCs during the initial phases of AD development. Moreover, the cytokine accumulation in RBCs seems to follow a faster accumulation kinetics compared to that elicited in the cerebral cortex.

## 4. Discussion

In the present study, the brain accumulation of neurodegeneration-related proteins such as homo- and heteroaggregates was analysed and compared to the levels of the same proteins in RBCs, using a validated animal model of AD at different stages of disease progression. Moreover, the amounts of IL-1*β*, chosen as a marker of AD-associated inflammation, were analysed both in brain tissues and RBCs. The main conclusions of this work are as follows: (a) brain A*β*, tau, and p-tau were elevated in SAMP8 mice compared to control and increased with AD progression; similar, although slower, accumulation kinetics were found in RBCs; (b) *α*-syn and its heterocomplexes, *α*-syn-A*β* and *α*-syn-tau, showed different accumulation kinetics in brain tissues and RBCs; and (c) both brain and peripheral IL-1*β* levels were elevated in SAMP8 mice, but increased earlier in RBCs. Overall, these results support RBCs as a valuable model to monitor neurodegeneration, suggesting A*β*, tau, and p-tau levels as suitable AD biomarkers in peripheral cells, both for diagnosis and follow-up.

Recent findings have suggested that each ND is not associated with the misfolding of a single specific protein, but rather with a mixed pattern of proteinopathies [4, 5], which reach peripheral compartments even years before the onset of clinical symptoms. Herein, a validated animal model of AD, i.e., SAMP8, and its control strain SAMR1 were used to quantify the accumulation of A*β*, tau, *α*-syn in brain tissues and RBCs and to compare them at different stages of progress of the disease.

Behavioural tests showed that SAMP8 mice develop an age-dependent deficit in spatial learning and memory performance, as compared with control SAMR1 mice, endorsing the validity of this animal model.

Consistent with previous data [1], A*β* was found to accumulate in the brain of AD animals. Similarly, A*β* levels were augmented in RBCs along with AD progression. This observation is of interest since A*β* levels in RBCs have been shown to increase with ageing in humans and to decrease upon providing an antioxidant supplement [15]. A noteworthy aspect we observed is a slower A*β* accumulation kinetics in RBCs compared to that detected in the brain. In this respect, A*β* increase in the brain and the onset of NDs have been linked to blood-derived A*β*, which can enter the brain [47], even preceding the neuronal and glial pathological alterations of AD brains [48]. Nevertheless, this “peripheral sink hypothesis” is still a bone of contention because inhibiting the A*β*-producing enzyme in the periphery does not alter A*β* accumulation in the CNS [49].

Similarly to what was observed for A*β*, tau and its phosphorylated form accumulated in the brain with ageing and/or AD progression in the SAM model, as previously reported by others [50, 51]. In particular, our data are consistent with evidences that tau hyperphosphorylation is an integral part of ageing and represents an early event in AD animal models [30, 52]. A similar, albeit quite slower, accumulation kinetics of tau and p-tau was found in the RBCs of SAMs, suggesting this protein as a valuable RBC marker reflecting brain neuropathology. Consistent with this hypothesis, significantly elevated concentrations of p-tau have been shown in the RBCs of PD patients [11].

Overall, our data allow hypothesising that the increase in the levels of A*β*, tau, and p-tau in the brain is due to an enhanced production in neurons associated with the progression of AD. In this respect, the increase of the same proteins in RBCs might result from an alteration of the blood-brain barrier (BBB), leading to the subsequent leakage of proteins from the brain to the blood. Therefore, further investigations are needed to elucidate the degree of production of these proteins in blood cells and the efficiency of proteasome systems deputed to the degradation of misfolded proteins.

Altered levels of *α*-syn, a protein commonly associated with PD, have recently been detected in the brain and CSF of AD patients [53]. In the present study, *α*-syn levels in the brain were found to be higher than those of age-matched controls in the early analysed phase of AD progression, consistent with the increase in the expression of *α*-syn found in 5-month SAMP8 [52]. Nevertheless, total *α*-syn was found to decrease with AD progression and comparatively with controls when 8-month-old mice were examined. The latter results are consistent with several findings reporting a significant decrease in the total concentration of brain *α*-syn in PD [54] and PD with dementia [55]. This partial controversy in *α*-syn levels in the early phase of AD suggests that SAMP8 does not fully reflect the human brain pathology associated with *α*-syn. Of note, a different trend was found for *α*-syn in RBCs: indeed, RBCs from SAMP8 showed significantly higher total *α*-syn concentrations than age-matched controls. These results are at odds with the data obtained in human RBCs from PD patients presenting lower levels than HC [11, 20] and highlight the need for further investigations to elucidate putative differences in posttranslational *α*-syn modifications, degradation pathways, and passage through the BBB [56, 57].

In addition to homoaggregates, the presence of heterocomplexes of *α*-syn (*α*-syn-tau and *α*-syn-A*β*) has been documented both in cellular models and patients' brains [5, 8, 9, 11, 58]. On this basis, *α*-syn heterocomplexes were measured for the first time in this experimental model through “homemade” immunoenzymatic assays. Interestingly, *α*-syn was shown to form heterocomplexes with A*β* and tau both in brain tissues and RBCs of SAM mice, similarly to previous data reported in human samples [5, 11, 22].

Both *α*-syn-A*β* and *α*-syn-tau concentrations in SAM brains followed the same accumulation kinetics shown for total *α*-syn. Indeed, the initial increment was followed by a decrement in the second phase of AD compared to controls and AD progression. Interestingly, *α*-syn-A*β* and *α*-syn-tau levels in RBCs displayed a similar trend, presenting a significant increase in AD mice compared to controls and along with AD progression. As observed for brain samples, *α*-syn heterocomplexes showed the same temporal kinetics of accumulation of total *α*-syn in RBCs, too. Consistent with these data, *α*-syn-A*β* concentrations in human RBCs were elevated in PD patients and correlated with the progress of the disease [11]. Surprisingly, our recent findings have probed a significant decrease of *α*-syn heterocomplexes in RBCs from AD patients [10]. These findings point out the need for further investigations in relation to the specific neurodegenerative disease and the fluid compartment in which these proteins are analysed.

With these data at our disposal, the accumulation of *α*-syn and its heterocomplexes in RBCs does not seem to reflect the brain trend. The discrepancy could be ascribed to the trafficking across the BBB or to the difficulty of measuring appropriately the *α*-syn oligomeric form [5].

In the present study, the possible relationship between AD progression and inflammation was investigated by assessing IL-1*β* levels, as one of the most important inflammatory mediators, both in brain tissues and RBCs from SAMP8 and SAMR1. In this sense, it has to be underlined that peripheral and central cytokines are released upon inflammation [59] and not strictly related to AD. Nevertheless, IL-1*β* has been demonstrated to be significantly higher in AD with respect to controls in both humans and animals [27–29] and can be considered as a good marker of inflammation in an animal model of this disease. In our hands, brain IL-1*β* levels increased in an AD animal model at 8 months and in age-matched control, as previously reported by others in the hippocampus [26]. Interestingly, IL-1*β* concentrations were higher in RBCs than in the brain. However, not surprisingly, RBCs have been identified recently as a reservoir for cytokines, with a median concentration 12-fold higher than plasma [18]. RBC IL-1*β* levels were significantly elevated in the pathological animals, consistent with the greater level of oxidative stress found in RBCs from SAMP8 [60]. These changes were particularly evident at 6 months, suggesting that the cytokine accumulation in RBCs seems to follow a faster kinetics compared to the cerebral cortex, and thus it could represent an early diagnostic for its early stage. In this respect, recent findings have highlighted a close link between peripheral inflammation and accumulation of misfolded proteins [11, 61]. For instance, inflammation has been shown to augment A*β* levels through an increased production in the brain, an increased influx, and a decreased efflux, due to alterations of the BBB [62]. Recently, peripheral inflammation has been shown to modulate the amyloid phenotype in mice by regulating blood-derived and local brain inflammatory cell populations involved in A*β* clearance [63]. On the other side, in animal models, in AD and in aged brains, accumulation of A*β* seems to trigger inflammatory responses with an enhanced production of inflammatory factors, which have been localised in brain plaques [64]. Besides the inflammasome pathway, amyloid fibrils have been proven to induce the secretion of proinflammatory cytokines through the activation of the Toll-like receptor 2 [65]. Future studies will investigate the involvement of this innate immune receptor in immune responses associated to amyloidosis in an AD animal model. Among the limitations of our study, it should be underlined that the available immunoenzymatic assays cannot distinguish the oligomeric or monomeric nature of ND-related proteins, both in brain tissues and blood. Further studies will confirm the multiaggregation status of these proteins.

In conclusion, we proved in the present study that A*β*, tau, and p-tau kinetics of accumulation in RBCs from SAMP8 followed similar patterns to those in the brain, suggesting these proteins as putative peripheral biomarkers of AD. Future studies will be needed to confirm our preliminary data.

## Figures and Tables

**Table 1:** Concentrations of A*β*, *α*-syn, tau, p-tau, *α*-syn-A*β*, and *α*-syn-tau in brain tissues and RBCs of SAMP8 and SAMR1 at different ages.

Brain	A*β* (ng/*μ*g)	*α*-Syn (ng/*μ*g)	Tau (ng/*μ*g)	p-Tau (pg/*μ*g)	*α*-Syn-A*β* (ng/*μ*g)	*α*-Syn-tau (ng/*μ*g)
SAMR1 6 m	7.63 ± 3.06	6.22 ± 4.59	7.51 ± 3.10	158 ± 92.2	1.35 ± 1.36	2.08 ± 1.58
SAMP8 6 m	13.5 ± 4.75^∗^	15.5 ± 7.73^∗^	7.28 ± 2.77	327 ± 118^∗^	2.70 ± 1.77	8.74 ± 3.12^∗∗^
SAMR1 8 m	8.81 ± 3.72	8.54 ± 5.94	13.0 ± 3.6^#^	123 ± 105	2.81 ± 1.68	1.54 ± 1.04
SAMP8 8 m	15.5 ± 4.8^∗^	0.69 ± 0.42^∗∗^^##^	22.1 ± 6.0^∗^^##^	397 ± 152^∗∗∗^	0.53 ± 0.50^∗^^#^	1.17 ± 1.49^###^
RBCs	A*β* (ng/mg)	*α*-Syn (ng/mg)	Tau (ng/mg)	p-Tau (ng/mg)	*α*-Syn-A*β* (ng/mg)	*α*-Syn-tau (ng/mg)
SAMR1 6 m	147 ± 48.1	37.5 ± 25.2	14.7 ± 1.59	0.36 ± 0.09	19.7 ± 13.6	3.56 ± 2.77
SAMP8 6 m	121 ± 68.6	279 ±242^∗∗∗^	12.1 ± 6.95	0.36 ± 0.09	39.9 ± 23.8^∗^	4.78 ± 6.04
SAMR1 8 m	123 ± 13.5	39.1 ± 47.4	14.0 ± 6.38	0.92 ± 0.74^##^	40.7 ± 50.7^#^	3.70 ± 3.57
SAMP8 8 m	213 ± 120^∗^^#^	204 ± 186^∗∗^	34.4 ± 23.9^∗^^#^	1.21 ± 1.33^##^	103 ± 99.4^#^	8.67 ± 5.45^∗^^##^

Values are expressed as mean ± SD. Differences among the groups were evaluated by a nonparametric analysis (Kruskal-Wallis). ^∗^*P* < 0.05, ^∗∗^*P* < 0.01, and ^∗∗∗^*P* < 0.001 indicate significant differences of SAMR1 versus the age-matched SAMP8. ^#^*P* < 0.05, ^##^*P* < 0.01, and ^###^*P* < 0.001 indicate significant differences between SAMR1 6 m and SAMR1 8 m or between SAMP8 6 m and SAMP8 8 m.

## Data Availability

The data used to support the findings of this study are available from the corresponding author upon request.

## Abbreviations

AD: Alzheimer's disease
ND: Neurodegenerative disease
A*β*: *β*-Amyloid
p-tau: Phospho-tau
*α*-syn: *α*-Synuclein
NTs: Neurofibrillary tangles
RBCs: Red blood cells
SAMP8: Senescence-accelerated mouse-prone
SAMR1: Senescence-accelerated mouse-resistant
IL-1*β*: Interleukin-1*β*.

## References

[1] Morley J. E., Armbrecht H. J., Farr S. A., Kumar V. B. (2012). The senescence accelerated mouse (SAMP8) as a model for oxidative stress and Alzheimer’s disease. *Biochimica et Biophysica Acta (BBA) – Molecular Basis of Disease*.

[2] Calsolaro V., Edison P. (2016). Neuroinflammation in Alzheimer’s disease: current evidence and future directions. *Alzheimer’s & Dementia*.

[3] Daniele S., Giacomelli C., Martini C. (2018). Brain ageing and neurodegenerative disease: the role of cellular waste management. *Biochemical Pharmacology*.

[4] Baldacci F., Lista S., Garaci F., Bonuccelli U., Toschi N., Hampel H. (2016). Biomarker-guided classification scheme of neurodegenerative diseases. *Journal of Sport and Health Science*.

[5] Giacomelli C., Daniele S., Martini C. (2017). Potential biomarkers and novel pharmacological targets in protein aggregation-related neurodegenerative diseases. *Biochemical Pharmacology*.

[6] Larson M. E., Sherman M. A., Greimel S. (2012). Soluble *α*-synuclein is a novel modulator of Alzheimer’s disease pathophysiology. *The Journal of Neuroscience*.

[7] Crews L., Tsigelny I., Hashimoto M., Masliah E. (2009). Role of synucleins in Alzheimer’s disease. *Neurotoxicity Research*.

[8] Andersen A. D., Binzer M., Stenager E., Gramsbergen J. B. (2017). Cerebrospinal fluid biomarkers for Parkinson’s disease – a systematic review. *Acta Neurologica Scandinavica*.

[9] Sengupta U., Guerrero-Muñoz M. J., Castillo-Carranza D. L. (2015). Pathological interface between oligomeric alpha-synuclein and tau in synucleinopathies. *Biological Psychiatry*.

[10] Baldacci F., Daniele S., Piccarducci R. (2019). Potential diagnostic value of red blood cells *α*-synuclein heteroaggregates in Alzheimer’s disease. *Molecular Neurobiology*.

[11] Daniele S., Frosini D., Pietrobono D. (2018). *α*-Synuclein heterocomplexes with *β*-amyloid are increased in red blood cells of Parkinson’s disease patients and correlate with disease severity. *Frontiers in Molecular Neuroscience*.

[12] Koric L., Guedj E., Habert M. O. (2016). Molecular imaging in the diagnosis of Alzheimer’s disease and related disorders. *Revue Neurologique*.

[13] Marksteiner J., Hinterhuber H., Hinterhuber C. (2007). Cerebrospinal fluid biomarkers for diagnosis of Alzheimer’s disease: beta-amyloid(1-42), tau, phospho-tau-181 and total protein. *Drugs Today*.

[14] Stolp H. B., Dziegielewska K. M. (2009). Review: Role of developmental inflammation and blood–brain barrier dysfunction in neurodevelopmental and neurodegenerative diseases. *Neuropathology and Applied Neurobiology*.

[15] Kiko T., Nakagawa K., Satoh A. (2012). Amyloid *β* levels in human red blood cells. *PLoS One*.

[16] Singh S. (2015). Antioxidants as a preventive therapeutic option for age related neurodegenerative diseases. *Therapeutic Targets for Neurological Diseases*.

[17] Arbos K. A., Claro L. M., Borges L., Santos C. A. M., Weffort-Santos A. M. (2008). Human erythrocytes as a system for evaluating the antioxidant capacity of vegetable extracts. *Nutrition Research*.

[18] Karsten E., Breen E., Herbert B. R. (2018). Red blood cells are dynamic reservoirs of cytokines. *Scientific Reports*.

[19] Eisele Y. S., Obermüller U., Heilbronner G. (2010). Peripherally applied A*β*-containing inoculates induce cerebral *β*-amyloidosis. *Science*.

[20] Wang X., Yu S., Li F., Feng T. (2015). Detection of *α*-synuclein oligomers in red blood cells as a potential biomarker of Parkinson’s disease. *Neuroscience Letters*.

[21] Daniele S., Costa B., Pietrobono D. (2018). Epigenetic modifications of the*α*-synuclein gene and relative protein content are affected by ageing and physical exercise in blood from healthy subjects. *Oxidative Medicine and Cellular Longevity*.

[22] Daniele S., Pietrobono D., Fusi J. (2018). *α*-Synuclein aggregates with *β*-amyloid or tau in human red blood cells: correlation with antioxidant capability and physical exercise in human healthy subjects. *Molecular Neurobiology*.

[23] Butterfield D. A., Poon H. F. (2005). The senescence-accelerated prone mouse (SAMP8): a model of age-related cognitive decline with relevance to alterations of the gene expression and protein abnormalities in Alzheimer’s disease. *Experimental Gerontology*.

[24] Griffin W. S. T., Sheng J. G., Roberts G. W., Mrak R. E. (1995). Interleukin-1 expression in different plaque types in Alzheimer’s disease: significance in plaque evolution. *Journal of Neuropathology & Experimental Neurology*.

[25] Patterson P. H. (1995). Cytokines in Alzheimer’s disease and multiple sclerosis. *Current Opinion in Neurobiology*.

[26] Tha K. K., Okuma Y., Miyazaki H. (2000). Changes in expressions of proinflammatory cytokines IL-1*β*, TNF-*α* and IL-6 in the brain of senescence accelerated mouse (SAM) P8. *Brain Research*.

[27] Lai K. S. P., Liu C. S., Rau A. (2017). Peripheral inflammatory markers in Alzheimer’s disease: a systematic review and meta-analysis of 175 studies. *Journal of Neurology, Neurosurgery & Psychiatry*.

[28] Gomaa A. A., Makboul R. M., el-Mokhtar M. A., Abdel-Rahman E. A., Ahmed I. A., Nicola M. A. (2019). Terpenoid-rich *Elettaria cardamomum* extract prevents Alzheimer-like alterations induced in diabetic rats via inhibition of GSK3*β* activity, oxidative stress and pro-inflammatory cytokines. *Cytokine*.

[29] Ebenezer I. S., Tite R. (1994). Sex difference in the feeding responses of non-deprived rats to the 5-HT1A agonists 8-OH-DPAT and gepirone. *Methods and Findings in Experimental and Clinical Pharmacology*.

[30] Canudas A. M., Gutierrez-Cuesta J., Rodríguez M. I. (2005). Hyperphosphorylation of microtubule-associated protein tau in senescence-accelerated mouse (SAM). *Mechanisms of Ageing and Development*.

[31] El-Agnaf O. M. A., Salem S. A., Paleologou K. E. (2006). Detection of oligomeric forms of *α*-synuclein protein in human plasma as a potential biomarker for Parkinson’s disease. *The FASEB Journal*.

[32] Pesini P., Pérez-Grijalba V., Monleón I. (2012). Reliable measurements of the *β-*amyloid pool in blood could help in the early diagnosis of AD. *International Journal of Alzheimer's Disease*.

[33] Zappelli E., Daniele S., Abbracchio M., Martini C., Trincavelli M. (2014). A rapid and efficient immunoenzymatic assay to detect receptor protein interactions: G protein-coupled receptors. *International Journal of Molecular Sciences*.

[34] Fumagalli M., Bonfanti E., Daniele S. (2015). The ubiquitin ligase Mdm2 controls oligodendrocyte maturation by intertwining mTOR with G protein**‐**coupled receptor kinase 2 in the regulation of GPR17 receptor desensitization. *Glia*.

[35] Emmanouilidou E., Elenis D., Papasilekas T. (2011). Assessment of *α*-synuclein secretion in mouse and human brain parenchyma. *PLoS One*.

[36] Teich A. F., Patel M., Arancio O. (2013). A reliable way to detect endogenous murine *β*-amyloid. *PLoS One*.

[37] Mandal P. K., Pettegrew J. W., Masliah E., Hamilton R. L., Mandal R. (2006). Interaction between A*β* peptide and *α* synuclein: molecular mechanisms in overlapping pathology of Alzheimer’s and Parkinson’s in dementia with Lewy body disease. *Neurochemical Research*.

[38] Hou X. Q., Song H. P., Chen Y. B. (2018). Effects of Bushen-Yizhi formula on age-related inflammation and oxidative stress in senescence-accelerated mice. *Molecular Medicine Reports*.

[39] Xu P., Xu S. P., Wang K. Z. (2016). Cognitive-enhancing effects of hydrolysate of polygalasaponin in SAMP8 mice. *Journal of Zhejiang University-Science B*.

[40] Kang L., Li S., Xing Z. (2014). Dihydrotestosterone treatment delays the conversion from mild cognitive impairment to Alzheimer’s disease in SAMP8 mice. *Hormones and Behavior*.

[41] Cerf E., Sarroukh R., Tamamizu-Kato S. (2009). Antiparallel *β*-sheet: a signature structure of the oligomeric amyloid *β*-peptide. *Biochemical Journal*.

[42] Kostylev M. A., Kaufman A. C., Nygaard H. B. (2015). Prion-protein-interacting amyloid-*β* oligomers of high molecular weight are tightly correlated with memory impairment in multiple Alzheimer mouse models. *The Journal of Biological Chemistry*.

[43] Buée L., Bussière T., Buée-Scherrer V., Delacourte A., Hof P. R. (2000). Tau protein isoforms, phosphorylation and role in neurodegenerative disorders. *Brain Research Reviews*.

[44] Santpere G., Puig B., Ferrer I. (2006). Low molecular weight species of tau in Alzheimer’s disease are dependent on tau phosphorylation sites but not on delayed post-mortem delay in tissue processing. *Neuroscience Letters*.

[45] Bartels T., Choi J., Kim N., Selkoe D. (2011). Non-denaturing purification of alpha-synuclein from erythrocytes. *Protocol Exchange*.

[46] Jacobi K. E., Wanke C., Jacobi A., Weisbach V., Hemmerling T. M. (2000). Determination of eicosanoid and cytokine production in salvaged blood, stored red blood cell concentrates, and whole blood. *Journal of Clinical Anesthesia*.

[47] Bu X. L., Xiang Y., Jin W. S. (2018). Blood-derived amyloid-*β* protein induces Alzheimer’s disease pathologies. *Molecular Psychiatry*.

[48] Citron M., Vigo-Pelfrey C., Teplow D. B. (1994). Excessive production of amyloid beta-protein by peripheral cells of symptomatic and presymptomatic patients carrying the Swedish familial Alzheimer disease mutation. *Proceedings of the National Academy of Sciences of the United States of America*.

[49] Georgievska B., Gustavsson S., Lundkvist J. (2015). Revisiting the peripheral sink hypothesis: inhibiting BACE1 activity in the periphery does not alter *β*-amyloid levels in the CNS. *Journal of Neurochemistry*.

[50] Blennow K. (2017). A review of fluid biomarkers for Alzheimer’s disease: moving from CSF to blood. *Neurology and Therapy*.

[51] Pallàs M. (2012). Senescence-accelerated mice P8: a tool to study brain aging and Alzheimer’s disease in a mouse model. *ISRN Cell Biology*.

[52] Álvarez-García Ó., Vega-Naredo I., Sierra V. (2006). Elevated oxidative stress in the brain of senescence-accelerated mice at 5 months of age. *Biogerontology*.

[53] Shi M., Tang L., Toledo J. B. (2018). Cerebrospinal fluid *α*-synuclein contributes to the differential diagnosis of Alzheimer’s disease. *Alzheimer's & Dementia*.

[54] Tokuda T., Salem S. A., Allsop D. (2006). Decreased alpha-synuclein in cerebrospinal fluid of aged individuals and subjects with Parkinson’s disease. *Biochemical and Biophysical Research Communications*.

[55] Fathy Y. Y., Jonker A. J., Oudejans E. (2018). Differential insular cortex subregional vulnerability to *α*-synuclein pathology in Parkinson’s disease and dementia with Lewy bodies. *Neuropathology and Applied Neurobiology*.

[56] Bates C. A., Zheng W. (2014). Brain disposition of *α*-Synuclein: roles of brain barrier systems and implications for Parkinson’s disease. *Fluids and Barriers of the CNS*.

[57] Miranda H. V., Cássio R., Correia-Guedes L. (2017). Posttranslational modifications of blood-derived alpha-synuclein as biochemical markers for Parkinson’s disease. *Scientific Reports*.

[58] Iofrida C., Daniele S., Pietrobono D. (2017). Influence of physical exercise on *β*-amyloid, *α*-synuclein and tau accumulation: an in vitro model of oxidative stress in human red blood cells. *Archives Italiennes de Biologie*.

[59] Ren K., Torres R. (2009). Role of interleukin-1beta during pain and inflammation. *Brain Research Reviews*.

[60] Nogués M. R., Giralt M., Romeu M. (2006). Melatonin reduces oxidative stress in erythrocytes and plasma of senescence-accelerated mice. *Journal of Pineal Research*.

[61] Träger U., Tabrizi S. J. (2013). Peripheral inflammation in neurodegeneration. *Journal of Molecular Medicine*.

[62] Kempuraj D., Thangavel R., Selvakumar G. P. (2017). Brain and peripheral atypical inflammatory mediators potentiate neuroinflammation and neurodegeneration. *Frontiers in Cellular Neuroscience*.

[63] Paouri E., Tzara O., Kartalou G. I., Zenelak S., Georgopoulos S. (2017). Peripheral tumor necrosis factor-alpha (TNF-*α*) modulates amyloid pathology by regulating blood-derived immune cells and glial response in the brain of AD/TNF transgenic mice. *The Journal of Neuroscience*.

[64] Doens D., Fernández P. L. (2014). Microglia receptors and their implications in the response to amyloid *β* for Alzheimer’s disease pathogenesis. *Journal of Neuroinflammation*.

[65] Gustot A., Raussens V., Dehousse M. (2013). Activation of innate immunity by lysozyme fibrils is critically dependent on cross-*β* sheet structure. *Cellular and Molecular Life Sciences*.

